# Modern Use of Soft Foil

**Published:** 1903-01-15

**Authors:** G. S. Junkerman


### MODERN USE OF SOFT FOIL.

BY G. S. JUNKERMAN, M.D., D.D.S.

Read before the Ohio State Dental Society, December 3, 1902.

I assume no responsibility for the appearance of this paper before the society. If you have any missiles of disapproval to hurl please remember that I am intrenched behind the broad shoulders of the chairman of the committee and that he should be the receptacle of your projectiles. I am still wondering why I should have been asked to prepare a paper on such an old subject. In fact I am perplexed with two trains of thought in explanation. It may have been from a much to be commended sense of charity in the belief that I did not know anything else; or it may have been a symptom of an awakening on the part of the profession, an echo of the past, or a revival in the memory of the methods used by some of the best men the profession has ever known.

Method in dental science is a chained circle of limited size. We drop one link and take up another and must necessarily in an average life take up the same link more than once. Is a request by unanimous vote of the executive committee of this society for a paper on soft foil an indication that we have arrived again at the soft foil link in the chained circle ? Speaking from a long experience I trust that such is the case, because I feel assured that this soft foil link will be so influenced by the hard campaigning of hard foil that it will be molded into a new link to be known as the modern soft foil method which I wish to lay before you to-day.

If we exclude method of insertion and look only at quality it is very generally agreed that gold is the best filling material that we have. Those who have condemned gold on account of method have been blinded by ignorance of the very best quality which that material possesses. I believe that there is a physical difference between soft and hard foil (two terms that I shall use instead of cohesive and non-cohesive). Soft gold becomes cohesive by either heat or force. Hard gold becomes non-cohesive by either heat or force. If you mallet soft gold it becomes cohesive, just as cohesive gold over-malleted becomes non-cohesive. This principle was not known, at least to any great extent to former soft foil workers. They made their cylinders with accuracy and precision and packed them as a stone-mason or brick-layer builds an arch so that one would hold the other. They may possibly at times have induced cohesion in the soft foil mass, but they never worked with that end in view ; consequently the old method did not permit of the insertion of large contour fillings such as we can insert to-day with soft foil. If it is conceded, and it must be because it has been so proven, that soft gold can be made cohesive enough during the process of insertion, then you take away the last leg upon which the cohesive foil operator has to stand. He should be condemned to life-long servitude for the untold torture that he has inflicted upon humanity by his unending cohesive foil operation; and the amalgam worker should be his slave to make amends for the "queer" that he has been unloading upon the public.

It was formerly thought necessary to have a special set of instruments to manipulate soft foil. Such from a modern point of view is not only not necessary but to be condemned. Any plugger that can be used advantageously for hard foil is likewise adaptable for soft foil. If large plugger point is condemned for hard foil it is doubly so for soft foil. Any good set of cohesive foil pluggers makes a most desirable set for soft foil. No special care need be given to rolling the gold evenly. All that is necessary is that it should be in large enough or small enough lumps to fit the receptacle into which it is to be placed. Good condensation with a mallet or hand pressure is required. It is not necessary or advisable to cut deep grooves, or retaining pits in order to have the cavity well prepared. General good form to the cavity is all that is required with the orifice just a slight degree smaller than the base. It requires more skill, but much less labor. Ninety-five percent of all fillings inserted can be inserted with soft foil. It is a humane method much less rasping and painful and requires so little time that it is preferred by our patients over all other filling materials. It may be truly said that in proportion as teeth need saving soft gold is the very best filling material that we have.

It might be of some interest to know just what fillings can be inserted and what ones are to be excluded from this method. It is probably not safe to attempt to restore the cutting edges of incisors. I have accomplished this feat, but it is of doubtful stability and endurance. It is usually necessary to have two walls between which to build. Not so much for the purpose of retention, but as a matrix which will permit of sufficient force being brought to bear upon the mass to insure cohesion. The two walls form the main matrix, but each piece of gold is used to form a matrix in the mass into which the next piece is to be placed. It might make it more clear to state, that but little attention is paid to the piece that you are inserting, but all attention to the formation of the matrix or the wedge shaped space for the insertion of the next piece. This interlacing of gold is best accomplished with fine instruments. The larger ones will cause bridging and will fail to induce the necessary cohesion. The condensation is lateral as well as vertical or directly in front of the plugger. The lateral condensation does not occur however, from pressure on the part of the plugger, but rather from the piece that you are inserting and on account of the wedge shaped space into which you are placing it. The question would arise, do you get as much cohesion as if the gold were annealled? You do not, but you get sufficient for the purpose and any more would be wasted strength. If you should crack off with a hammer, the tooth from around one of these fillings you would generally find the filling in one mass and while it could be separated more easily than cohesive gold, yet the cohesion is quite considerable and sufficient. Centrals, laterals and canines with buccal and labial walls gone, can be easily restored with soft foil. The approximal cavities in molars and bicuspids with a fraction of the lateral walls remaining can be perfectly restored and we might say, every cavity with the exception of the cutting edges becomes easy not alone to the operator, but to the patient. Humanity cries out for operations that are speedy and scientific. It is not the dentist who makes a perfect operation who can claim skill, if at the same time he exhausts the vital energy of his patient. One may be able to cut well, but the skillful surgeon does all that and saves the blood of his patient too. It is not alone to the accurate, but to the speedy and accurate that the appelation of good dentist should be applied.

DISCUSSION.

Dr. H. L. Ambler: I have no doubt the essayist can do what he claims; but I have never been able to make non-cohesive gold cohesive by malleting it. I suppose when he speaks of soft gold he means non-cohesive gold, and when he speaks of hard gold he refers to cohesive gold. The first cohesive gold was made in 1839 by James Leslie of Cincinnati. It was called cohesive because it became so by annealing. Before that all golds were supposed to be non-cohesive, and it was thought to be a great gain to have the gold so non-cohesive that it worked soft like lead. It is not generally supposed that this non-cohesive foil can be made cohesive by force as in malleting. Dr. Black made a set of experiments in which fillings were made with cohesive and non-cohesive foils, and then tested for specific gravity, strength, etc. It was found that fillings made wholly of soft foil contained more air space and were capable of more condensation that those made of various combinations of non-cohesive and cohesive foils, and the more cohesive foil used, the less condensation was possible. These experiments clearly showed that where strength is demanded non-cohesive foil can not be depended upon. According to the testimony of one of our best gold foil makers there can be no physical union between the layers of non-cohesive foil, it is entirely of a mechanical character. This was well recognized by soft foil operators years ago and the principle of wedging the gold into the cavity was universally adopted. Contour filling, such as are now made of cohesive foil were seldom attempted by old time operators.

I believe many cavities may be well filled with non-cohesive gold foil. Where the walls are irregular, with deep under cuts and the contour of the tooth is intact, good fillings may be made. These fillings when made in the occlusal surfaces often wear out by the force of mastication. But in these same cavities I believe that a good cohesive foil properly annealed will make a much more perfect and also durable filling. I have tested many of the so-called non-cohesive gold foils and have yet to find one that can not be made cohesive by proper annealing; but I do not think a non-cohesive gold can be made cohesive by force. Where this gold is actually non-cohesive it is an impure gold. The process of making non-cohesive gold is a manufacturer's secret. Foils that have been rendered non-cohesive by subjecting them to the fumes of ammonia, sulphur, phosphorus, etc., can be easily converted to cohesive foils by proper annealing.

Dr. E. M. Cook: I think we are too often confused by the terms cohesive and non-cohesive gold foils, and I amagine very few operators use strictly non-cohesive foil. The strictly non-cohesive foil has its place, and I sometimes get very good results from using. I do not think it can be made cohesive as the essayist says by malleting. Although I generally use it with the same pluggers I use in condensing cohesive gold. I can not see how even semi-cohesive gold can be made cohesive by malleting it.

Dr. J. A. Stephen: I don't like the terms "soft" and "hard" as used by the essayist, it is much better to retain the terms cohesive and non-cohesive. I use cohesive and non-cohesive gold in combination to good advantage by placing a layer of the non-cohesive gold on the cavity margins and covering it with a layer of cohesive foil. By placing it in alternate layers a hard filling will be made and at the same time the adaptation to the cavity walls is made more perfect.

Dr. Junkerman: In closing this discussion I would say that it is possible to make non-cohesive gold adhere into a solid filling, simply by working it with proper filling instruments. I have done this with Abbey's non-cohesive foil unannealed, and in the presence of several gentlemen who were well qualified to decide whether I had succeeded or not. I think I could teach Dr. Ambler to do the same. By heating gold foil we change its molecular condition and produce a re-adjustment of its crystaline character which favors cohesive or physical union. I am not prepared to say that a similar change takes place under the manipulative process of packing the gold into the tooth cavity; but I am very sure that some change takes place which favors an inseperable union. I have tested for tenacity fillings made in this manner and find them sufficient to withstand any probable strain. In reality cohesive gold is more brittle than non-cohesive. I have here some samples of fillings made of non-cohesive gold according to my method, and you never saw more compact and polished surfaces on any fillings.

